# Copper resistance selection and activity changes of antioxidases in the flesh fly *Boettcherisca peregrina*

**DOI:** 10.1093/jis/14.1.116

**Published:** 2014-09-01

**Authors:** Guoxing Wu, Xi Gao, Jiaying Zhu, Cui Hu, Gongyin Ye, Nannan Liu

**Affiliations:** 1 State Key Laboratory of Rice Biology, Key Laboratory of Agricultural Entomology, Ministry of Agriculture, Institute of Insect Sciences, Zhejiang University, Hangzhou 310058, China; 2 College of Plant Protection, Yunnan Agricultural University, Kunming 650201, China; 3 Key Laboratory of Forest Disaster Warning and Control of Yunnan Province and Key Laboratory of Southwest Mountain Forest Resources Conservation and Utilization of Ministry of Education, Southwest Forestry University, Kunming 650224, China

**Keywords:** superoxide dismutase, catalase, glutathione S-transferase, development, reproduction

## Abstract

Natural populations of *Boettcherisca* ( *Sarcophaga* ) *peregrina* Robineau-Desvoidy (Diptera: Sarcophagidae) were maintained for 20 generations and reared either on unpolluted diet or on polluted diet containing copper at a median lethal concentration (LC _50_ ) determined every five generations. This resulted in two reliable strains: the relative susceptible strain (S) and the copperresistant strain (R). The metal accumulation, growth and development, reproduction, and antioxidant enzymes were analyzed in the two strains. The results showed that compared with the S strain, the R strain showed increased metal accumulation and fecundity of female adults. Regardless of whether larvae were fed on diet with or without Cu ^2+^ , the R strain showed higher activity of superoxide dismutase and glutathione S-transferase than the S strain, although without statistical significance. Moreover, the activity of superoxide dismutase and glutathione S-transferase increased when *B. peregrina* larvae were exposed to Cu ^2+^ at 100 µg/g but decreased when they were exposed to Cu ^2+^ at 800 µg/g. Larval catalase activity in the R strain was higher than in the S strain when larvae were fed on diet with or without Cu ^2+^ , although these differences were significant only at the 100 µg/g concentration. Moreover, the activity of catalase decreased when larvae were exposed to experimental Cu ^2+^ . Beyond all expectations, larval glutathione reductase activity was not significantly different between the two strains but changed slightly when larvae were exposed to experimental Cu ^2+^ . These results indicate that copper resistance in *B. peregrina* larvae is mediated by superoxide dismutase, catalase, and glutathione S-transferase. These results also help in establishing a physiological link between antioxidase activity and the resistance level of *B. peregrina* to copper.

## Introduction

Heavy metal pollution has become a global environmental problem and severely threatens biological diversity and human health. Because insects form an important group with global biological diversity ( Sun et al *.* 2007 ), much attention has been paid to the potential effects of heavy metal pollution on insects. One of the important indicators of heavy metal pollution in insects are antioxidases, such as superoxide dismutase (SOD), catalase, peroxidase, glutathione peroxidase (GSH-Px), glutathione S-transferase (GST), and glutathione reductase (GR), that remove reactive oxygen species generated by insects exposed to heavy metals ( Zamam et al. 1994 , Ahmad 1995 , Pardini 1995 , Migula and Glowacka 1996 , Stone et al *.* 2002 , Wilczek et al. 2003 , Li et al. 2005 , Wang et al *.* 2006 ). Like other heavy metals that are required in trace amounts to maintain homeostasis, copper is also one of the micronutrients essential for insects although an excess dietary intake of copper can be toxic in some circumstances. Many of the toxic effects of copper, such as increased lipid peroxidation in cell membranes and DNA damage, are related to its role in the generation of oxygen free radicals ( Kadiiska et al. 1992 , Bremner 1998 , Schümann et al. 2002 ). In insects, induction of reactive oxygen species by copper alters the activity of antioxidant enzymes ( Korsloot et al. 2004 , Migula et al. 2004 , Wang et al *.* 2006 ). Thus far, not much is known about the relationships between antioxidases and copper resistance levels in insects. Therefore, we developed a copperresistant strain of the flesh fly *Boettcherisca* ( *Sarcophaga* ) *peregrina* Robineau-Desvoidy (Diptera: Sarcophagidae) and compared the changes effected by exposure to Cu2+ between copperresistant and susceptible flesh fly strains. Flesh flies have been models to study various aspects of insects, such as physiology, biochemistry, development, and reproduction, among others. Larvae of these flies feed on carrion or feces and cause myiasis in livestock and humans ( Braverman et al. 1994 , Iqbal et al *.* 2011 ). *Boettcherisca peregrina* is one flesh fly species that has been studied widely because of its use in forensic entomology ( Sukontason et al *.* 2010 ). As a model insect, *B. peregrina* could explain the cytotoxic effects caused by metal pollution on insects. Previous studies have reported the effect of copper on the activity of SOD, catalase, and peroxidase ( Wang et al. 2006 ), the development and reproduction ( Wu et al. 2007 ), and the ultrastructure of midgut and Malpighian tubules ( Wu et al *.* 2009 ) in *B. peregrina* larvae. Here we report the selection of a copperresistant strain and changes in antioxidase activity in the flesh fly *B. peregrina* . These results will be helpful to understand the relationship between the activity of antioxidases and the level of copper resistance in *B. peregrina* .

## Materials and Methods

### Insects

*Boettcherisca peregrina* was maintained in an artificial climate chamber (25 ± 1°C, photoperiod of 14:10 L:D) for five years in the laboratory. Larvae were fed on wheat bran:water:porcine liver mixed at a ratio of 3:5:6, and adults were fed on water and sucrose.

### Toxicity determination

One-day-old flesh fly larvae were transferred to a glass vial containing 100 g artificial diet supplemented with the following Cu2+ concentrations: 50, 100, 200, 400, 800, 1,600, and 3,200 μg/g of artificial diet ( Wu et al *.* 2009 ). The control group was fed on artificial diet without Cu ^2+^ . Three replicates of about 30 larvae each were used for each Cu ^2+^ concentration and the control. The number of dead individuals in each treatment was counted when the larvae pupated. Regression equations, LC _50_ , and confidence interval were calculated by using a data processing system (DPS) for practical statistics ( Tang and Feng 2002 ).

### Selection of fly strains

One *B. peregrina* population fed on unpolluted diet was maintained in the laboratory for 20 generations (F _20_ ) and resulted in a copper-susceptible strain (S). A copperresistant strain (R) of *B. peregrina* was created by rearing one-day-old larvae on diet containing Cu ^2+^ at LC _50_ concentrations (median lethal concentration) determined every five generations. Individuals surviving the treatment were screened and used for the next generation. Such selection was continued for 20 generations, resulting in the R strain.

### Accumulation of Cu2+ in larvae and its effects on growth and development

Groups of 300 newly hatched larvae (within 8 hr) in the 20th generation (F _20_ ) were fed on diets containing Cu ^2+^ at concentrations of 0, 100, and 800 µg/g. Each group was reared in a glass bottle, and each concentration was replicated three times. After four days of treatment, 30 larvae from each group were picked randomly, washed with distilled water, and starved for 24 hr. They were then dried on paper towels and weighed on an electronic scale (AB204-E, Mettler Toledo, www.mt.com ). Each treatment was divided into four groups with one group of larvae treated with xylene:ethanol (1:1) solution to measure larval body length by using a vernier caliper after stretching. The second group of larvae was used to determine tissue metal content by using an atomic absorption spectrophotometer (AAnalyst100, Perkin Elmer, www.perkinelmer.com ) after digestion with 1 mL mixed acid (HClO _4_ :HNO _3_ =1:5 v/v) ( Wu et al *.* 2007 ). The third group was used to determine enzyme activity, and the remaining larvae in the fourth group were allowed to pupate, emerge, and mate to determine egg production by individual females.

### Enzyme activity measurement

To measure enzyme activity, larvae were first washed with the appropriate buffer solution, mixed with ice-cold buffer (1 mL buffer was added to 0.5 g larvae), and homogenized on ice. The homogenate was then centrifuged at 10,000 x *g* for 10 min at 4°C, and the supernatant was used as the enzyme preparation.

The SOD activity was determined as described previously by McCord and Fridovich (1969) and Deng and Yuan (1991) . Briefly, about 10 µL enzyme preparation was added to 4.5 mL Tris-HCl (50 mM; pH 8.2), mixed with 10 µL 45 mM pyrogallol, and homogenized immediately. The homogenate was transferred to a 1 cm cuvette to measure the optical density (OD) at 325 nm every 30 sec, maintaining the auto oxidation rate around 0.07 OD/min. One activity unit was defined as the amount of enzyme required to inhibit 50% auto oxidation in 1 min in 1 mL enzyme preparation. The SOD activity and specific activity were then calculated by using Equations [1] and [2].

${\rm{SOD\, activity (U/mL) = }}\frac{{\frac{{0.070 - \Delta {A_{325nm}}/\min }}{{0.070}} \times 100\% }}{{50\% }} \times {\rm{reaction\, volume}}\frac{{{\rm{dilution\, factor}}}}{{{\rm{sample\, volume}}}}$

[1]

${\rm{Specific\, activity (U/mg\, protein) = }}\frac{{SOD\,{\rm{activity}}(U/mL)}}{{{\rm{Protein\, concentration}}(mg/mL)}}$

[2]

Catalase activity was measured according to the method described by Barbehenn (2002) . The reaction solution contained 665 µL phosphate buffer (66 mM; pH 7.0), 25 µL enzyme preparation, and 10 µL 3% H _2_ O _2_ . The OD was measured continuously for 5 min every 30 sec at 240 nm _._ Catalase activity was expressed as the amount of H _2_ O _2_ reduced per mg protein in 1 min. The extinction coefficient was 39.4 M ^-1^ · cm ^-1^ ( Aebi 1984 ).

Activity of GR was measured by using Bergmeyer’s method ( Bergmeyer 1963 ) with slight modifications. Briefly, about 3 mL reaction mix was prepared that contained 0.1 mM phosphate buffer (pH 7.8), 1 mM Na _2_ EDTA, 1 mM oxidized glutathione (GSSG), 0.2 mM NADPH-Na _4_ , and 140 µL enzyme preparation. Absorbance at 340 nm was measured continuously for 5 min by using a UV spectrophotometer.

The GST was measured as described by Habig et al. (1974) . About 50 µL enzyme preparation was mixed with 1.93 mL 0.1 M phosphate buffer (pH 7.6) and 100 µL 0.05 M reduced glutathione, incubated at 25°C for 5 min, and then mixed with 20 µL 0.01 M 1-chloro-2,4-dinitrobenzene. The OD was then measured at 340 nm within 5 min. The extinction coefficient was 9.6 mM ^-1^ · cm ^-1^ .

### Data analysis

Data were analyzed by analysis of variance (ANOVA) in the DPS software ( Tang and Feng 2002 ) followed by Duncan’s multiple comparison method to compare within treatments. Levels of significance at *P* < 0.05 were considered as significant and at *P* < 0.01 as highly significant , whereas *P* > 0.05 was considered as not significant.

## Results

### Selection for copper resistance

To select copperresistant *B. peregrina,* larvae were fed on diet containing Cu ^2+^ at an LC _50_ concentration, which was determined once every five generations. Copper resistance in *B. peregrina* larvae developed slowly. For the R strain, LC _50_ values are listed in Table 1 . The LC _50_ in F _20_ of the R strain was only 1.64-fold higher than that in F _0_ and 1.68-fold higher than that in the S strain, which fed on unpolluted diet for 20 generations.

**Table 1.**
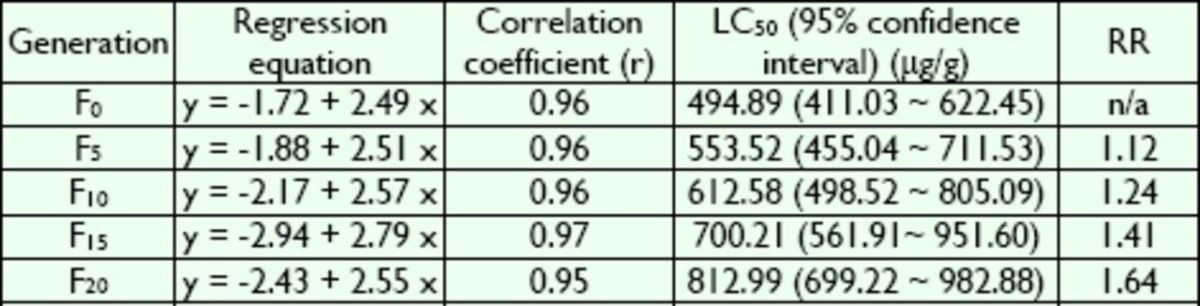
Resistance development of *B. peregrina* larvae to Cu ^2+^ .

RR values represent resistance rates at the LC50 relative to F0 generation; n/a, not applicable.

### Cu2+ accumulation in larvae

**Figure 1.**
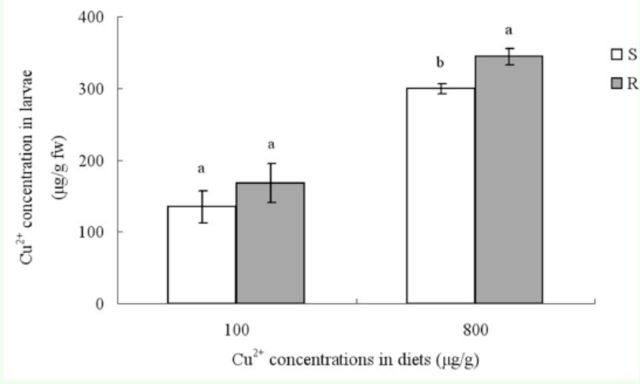
Cu ^2+^ accumulation in R and S strains of *B. peregrina* larvae. Values are means ± standard deviation. Same lowercase letters represent no significant difference after exposure to same concentration of Cu ^2+^ ( *P* < 0.05) and different letters represent significant differences ( *P* < 0.05) (Duncan’s multiple range test); fw, fresh weight; S, susceptible strain; R, copperresistant strain.

Larvae of the S and R strains fed on diet containing Cu ^2+^ at 800 µg/g accumulated more Cu ^2+^ than those fed on diet with Cu ^2+^ at 100 µg/g ( *P* < 0.01). On both diets, R-strain larvae accumulated more Cu ^2+^ than S-strain larvae. A significant difference in Cu ^2+^ accumulation was observed between R- and S-strain larvae fed on diet with Cu ^2+^ at 800 µg/g ( *P* < 0.01), but when they were fed on diet with Cu ^2+^ at 100 µg/g, the difference was not statistically significant ( *P >* 0.05) ( Fig. 1 ).Protein concentration was determined according to Bradford (1976) by using Coomassie Brilliant blue G _250_ . A standard curve was prepared with bovine serum albumin.

### Effects of Cu2+ on larval development

As shown in Tables 2 and 3 , the R and S strains were significantly different from each other in body weight ( *P* < 0.01) and length ( *P* < 0.01) when larvae were fed on Cu ^2+^ -free diet. After a four-day treatment with Cu ^2+^ , no significant difference in body weight ( *P* > 0.05) was observed between the two strains at 100 µg/g, but a significant difference was observed at 800 µg/g. However, the body lengths were signifi cantly different at both concentrations ( *P* < 0.01). Interestingly, body weight decreased at low concentrations of Cu ^2+^ (100 µg/g) in the S strain but increased slightly in the R strain. Similar body weights between the two strains at 100 µg/g ( *P* < 0.05) suggest the adaptation of the R strain to the low Cu ^2+^ concentration.

**Table 2.**
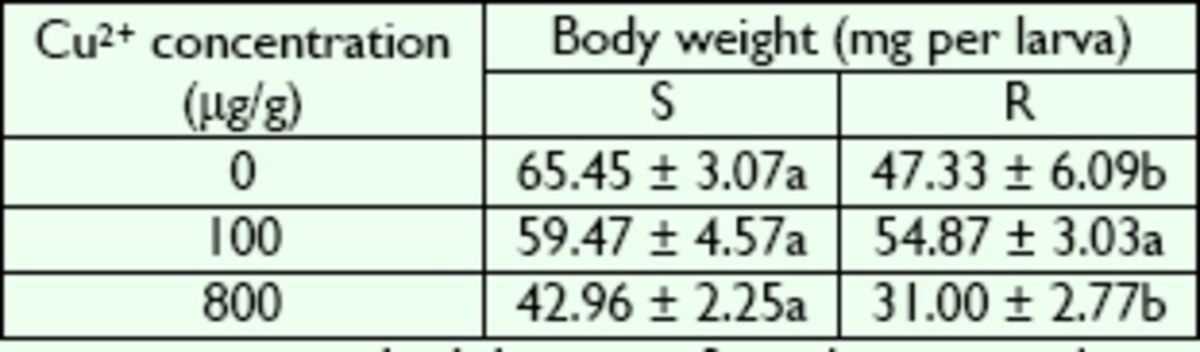
Body weight of R- and S-strain larvae of *B. peregrine* after exposure to Cu2+.

Values are means ± standard deviation. Same lowercase letters in a row represent no significant difference ( *P* > 0.05); different letters represent significant differences ( *P* < 0.05) (Duncan’s multiple range test ). S, susceptible strain; R, copperresistant strain.

**Table 3.**
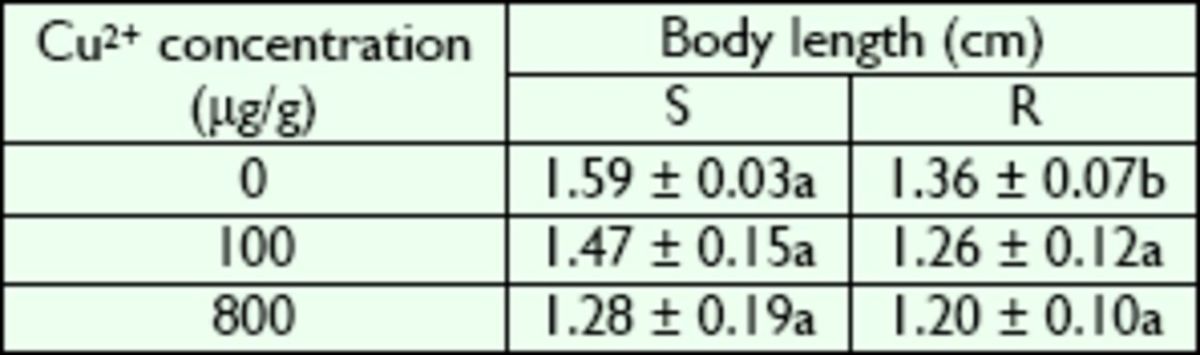
Body length of R- and S-strain larvae of *B. peregrine* after exposure to Cu2+.

Values are means ± standard deviation. Same lowercase letters in a row represent no significant difference ( *P* > 0.05); different letters represent significant differences ( *P* < 0.05) (Duncan’s multiple range test ) . S, susceptible strain; R, copperresistant strain.

### Effects of Cu2+ on adult reproduction

The R and S strains showed no significant difference in adult egg production ( *P* > 0.05) when larvae were fed on Cu ^2+^ -free diet ( Table 4 ). After Cu ^2+^ treatment during the larval stage, adult egg production declined significantly in the R and S strains at high Cu ^2+^ concentrations (800 µg/g) ( *P* < 0.01). However, treatment of larvae with low Cu ^2+^ concentrations (100 µg/g) did not cause a significant difference between the R and S strains ( *P* < 0.05).

**Table 4.**
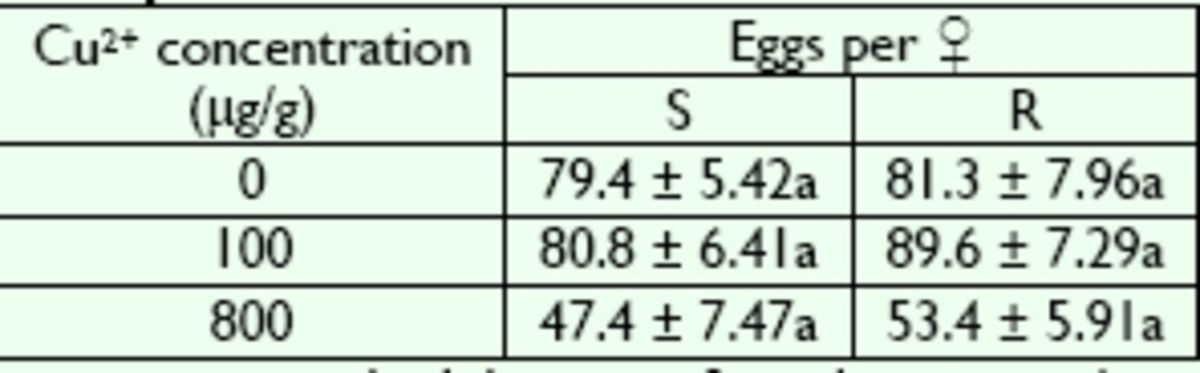
Adult female fecundity in R and S strains of *B. peregrine* after exposure to Cu2+.

### Effects of Cu2+ on larval enzyme activity

As shown in Table 5 , SOD activity of the R strain was higher than that of the S strain when larvae were fed on diet with or without Cu ^2+^ , although without statistical significance ( *P* > 0.05). The SOD activity was enhanced after larvae of both strains were continuously fed for four days on diet with Cu ^2+^ at 100 µg/g but was suppressed when larvae were fed on diet containing Cu ^2+^ at 800 µg/g.

**Table 5.**
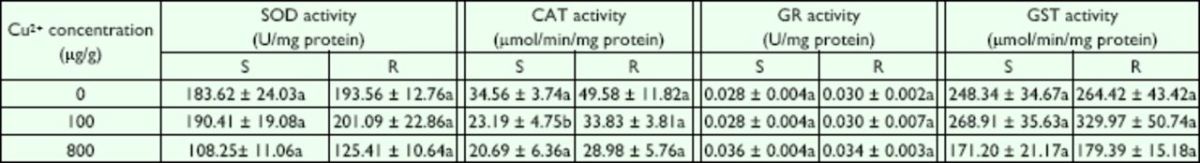
Enzyme activity in R and S strains of *B. peregrina* larvae.

Values are means ± standard deviation. Same lowercase letters in a row in an enzyme activity represent no significant difference *(P >* 0.05) and different letters represent significant differences *(P* < 0.05) (Duncan’s multiple range test). SOD, superoxide dismutase; CAT, catalase ; GR, glutathione reductase; GST, glutathione S-transferase; S, susceptible strain; R, copperresistant strain.

Larval catalase activity was higher in the R strain than in the S strain when larvae were fed on diet with or without Cu ^2+^ , although these differences were significant ( *P* < 0.05) only at the 100 µg/g concentration ( Table 5 ). After four days of Cu ^2+^ treatment, larval catalase activity was suppressed significantly in both strains ( *P* < 0.05) in a dose-dependent manner; the higher the Cu ^2+^ concentration the lower the catalase activity. At the low Cu ^2+^ concentration (100 µg/g), the activity of catalase was significantly different between the two strains ( *P* < 0.05), whereas at the high Cu ^2+^ concentration (800 µg/g), the catalase activity was not significantly different between the two strains ( *P* > 0.05).

Larval GR activity was similar in the R and S strains when larvae were fed on diet with or without Cu ^2+^ ( Table 5 ). Even after a four-day Cu ^2+^ treatment, larval GR activity in both strains showed no significant change ( *P* > 0.05).

Similar to SOD activity, larval GST activity was higher in the R strain than in the S strain when larvae were fed on diet with or without

Cu ^2+^ , but without statistical significance ( *P* > 0.05) ( Table 5 ). Compared with larval GST activity on Cu ^2+^ -free diet, GST activity in both strains increased after a four-day treatment with Cu ^2+^ at 100 µg/g, although there was no significant difference. However, after a four-day treatment with Cu ^2+^ at 800 µg/g, larval GST activity was significantly suppressed in the R and S strains ( *P* < 0.05) compared with their larval GST activity in the Cu ^2+^ -free treatment.

## Discussion

Changes in the activity of antioxidant enzymes are important to tolerate copper accumulation in insects ( Korsloot et al. 2004 , Migula et al *.* 2004 , Wang et al *.* 2006 ). However, such antioxidant enzyme activity has not been reported in relative copperresistant insect strains. In the present study, regardless of the presence or absence of Cu ^2+^ in the diet of *B. peregrina* larvae, the R strain had higher SOD, catalase, and GST activity than the S strain. Moreover, the activity of SOD and GST increased when *B. peregrina* larvae were exposed to Cu ^2+^ at 100 µg/g but decreased when larvae were exposed to Cu ^2+^ at 800 µg/g. Our results differ from those of Wang et al (2006) , who reported that the activity of SOD and catalase in *B. peregrina* was significantly inhibited with increasing Cu ^2+^ concentrations. These discrepancies support the notion that patterns in antioxidative enzyme activity are species specific and correlate to the levels of metal pollution or metal loads in the insect’s body ( Migula et al *.* 2004 ).

In contrast, larval GR activity was not significantly different between the R and S strains, and the activity of GR slightly changed when larvae were exposed to experimental Cu ^2+^ . This finding is similar to the reported GR activity in *Phyllobius betulae* F. (Coleoptera: Curculionidae) ( Migula et al *.* 2004 ).

At homeostatic conditions, SOD produces hydrogen peroxide by rapidly dismutating O _2_^•^ ‾ (2O _2_^•^ ‾ + 2H ^+^ →H _2_ O _2_ + O _2_ ) ( Richter and Schweizer 1997 , Wolin and Mohazzab-H 1997 ), which is a superoxide anion radical predominantly produced in the respiratory chain of mitochondria by auto oxidation of reduced components ( Winyard et al. 1994 ). In the presence of H _2_ O _2_ , which is an oxidizing environment, Cu ^2+^ reacts with reduced glutathione (GSH) to produce Cu ^+^ and a thiyl radical, GS ^•^ , which reacts with GS‾ to result in GSSG ^•^ ‾. The latter is a strongly reducing molecule that reacts rapidly with oxygen to yield O _2_^•^ ‾ ( Brouwer and Brouwer-Hoexum 1998 ). In our study, the increased SOD activity in *B. peregrina* indicates its critical role in converting O _2_^•^ ‾ into H _2_ O _2_ to mitigate the damaging effects exerted by excess O _2_^•^ ‾.

In *B. peregrina,* an increase in the amounts of H _2_ O _2_ also results in increased catalase activity, which is required to decompose H _2_ O _2_ ( Sohal et al. 1995 ). In general, H _2_ O _2_ is degraded to H _2_ O by two enzyme systems, catalase and glutathione peroxidase (GSH-Px) ( Korsloot et al. 2004 ), although differences between organisms have been observed. For example, GSH-Px plays an important role in mammals but is not present in nematodes and insects ( Orr and Sohal 1994 , Beckmann and Ames 1997 ). This indicates that the reaction H _2_ O _2_ + 2GSH → GSSG + 2H _2_ O catalyzed by GSH-Px and the GSH-regenerated reaction GSSG + NADPH + H ^+^ → 2GSH + NADP ^+^ catalyzed by GR do not occur in insects. This could explain the low GR activity that was not significantly different between the R and S strains of *B. peregrina* .

The enzyme GST plays an active role in the detoxification of endogenous and exogenous compounds and is ubiquitously distributed in the biota. Increased GST activity was reported in the carabid beetle *Pterostichus oblon-gopunctatus* F. (Coleoptera: Curculionidae) collected from metal-polluted areas ( Stone et al. 2002 ). In the western honey bee, *Apis mellifera* L. (Hymenoptera: Apidae), Smirle and Winston (1988) emphasized the role of GST in defense against the cytotoxic action of metals, and similar results were observed in cadmium-treated red wood ants *Formica aqui-lonia* Yarrow (Hymenoptera: Formicidae) ( Migula 1997 ). Changes in GST activity in the carabid beetle *Poecilus cupreus* L. also depended on the metals used and their doses to detoxify cadmium or zinc ( Wilczek et al. 2003 ). This holds true for GST activity in *B. peregrina* larvae fed on diet with Cu ^2+^ . Compared with the larval GST activity in the Cu ^2+^ -free treatment, GST activity in the R and S strains of *B. peregrina* increased after a four-day exposure to Cu ^2+^ at 100 µg/g and significantly decreased after a four-day exposure to Cu ^2+^ at 800 µg/g. Moreover, larval GST activity was higher in the R strain than in the S strain indicating that copper resistance in *B. peregrina* may be linked to GST activity.

In conclusion, results of the present study showed that increased resistance to Cu ^2+^ in the R strain resulted in enhanced fecundity and Cu ^2+^ accumulation compared with the S strain. Copper resistance in *B. peregrina* larvae was mediated by SOD, catalase, and GST rather than GR. Antioxidative enzyme activity was correlated to the levels of metal exposure or the metal loads in the body. These factors should therefore be considered in the design of experiments to investigate antioxidative enzyme activity in *B. peregrina* .
